# The rise of preprints in earth sciences

**DOI:** 10.12688/f1000research.133612.1

**Published:** 2023-05-30

**Authors:** Olivier Pourret, Daniel Enrique Ibarra

**Affiliations:** 1 UniLaSalle, AGHYLE, Beauvais, France; 2 2Department of Earth, Environmental Sciences, and Institute at Brown for Environment and Society, Brown University, Providence, Rhode Island, USA

**Keywords:** Open Access, Preprint, Open Science

## Abstract

The rate of science information's spread has accelerated in recent years. In this context, it appears that many scientific disciplines are beginning to recognize the value and possibility of sharing open access (OA) online manuscripts in their preprint form. Preprints are academic papers that are published but have not yet been evaluated by peers. They have existed in research at least since the 1960s and the creation of ArXiv in physics and mathematics. Since then, preprint platforms—which can be publisher- or community-driven, profit or not for profit, and based on proprietary or free and open source software—have gained popularity in many fields (for example, bioRxiv for the biological sciences). Today, there are many platforms that are either disciplinary-specific or cross-domain, with exponential development over the past ten years. Preprints as a whole still make up a very small portion of scholarly publishing, but a large group of early adopters are testing out these value-adding tools across a much wider range of disciplines than in the past. In this opinion article, we provide perspective on the three main options available for earth scientists, namely EarthArXiv, ESSOAr/ESS Open Archive and EGUsphere.

## Introduction

A research article’s preprint is its initial draft shared online, which is frequently (but not always) created before submission to a journal and formal peer review ( Sarabipour *et al*., 2019). Preprint archiving services have existed since the 1960s, and thus are not a recent invention ( Ginsparg, 2016). A centralized online network called arXiv, pronounced “är kv” (from the Greek letter "chi"), was created in August 1991 to exchange physics preprints ( Bourne *et al.*, 2017). For more than 30 years, arXiv has assisted the fields of physics, mathematics, and computer science, during which time the rate of scientific knowledge dissemination rapidly accelerated ( Ginsparg, 2016; Tennant *et al*. 2019).

A range of cross-domain or discipline-specific preprint platforms now exist, with exponential growth these last ten years ( Kirkham *et al*., 2020). Preprints as a whole only represent a very small fraction of scholarly publication, but a strong group of early adopters is starting to adopt their use, which is adding value across a much wider range of disciplines than before. Preprint archiving may aid in the modernization of Earth Sciences publishing by removing obstacles to widespread scientific engagement and stumbling blocks to the development of an open and transparent research culture ( Pourret *et al*., 2022).

In this Opinion Article, we further look at the evolution of three main options for earth scientists, namely EarthArXiv, ESSOAr/ESS Open Archive and EGUsphere and provide opinion on benefits and issues using preprints in earth sciences.

## Preprints in earth sciences

Preprints have recently gained popularity across a wider range of academic fields, including the Earth Sciences ( Nature Geoscience Editorial Board, 2018). The three main preprints servers in Earth Sciences are EarthArXiv, ESSOAr/ESS Open Archive and EGUsphere.

(i) EarthArXiv ( Narock *et al*., 2019) was created in 2018 and initially powered by OSF Preprints, and moved to a new infrastructure as a result of an emerging collaboration with California Digital Library in 2020.
(ii) ESSOAr that recently evolved in ESS Open Archive, was developed in a joint initiative by the American Geophysical Union with financial support from Wiley.
(iii) Earth Scientists who have published in the many journals of the European Geosciences Union (EGU) have already become accustomed to such openness and are posting their work prior to peer-review as a discussion on the Copernicus platform ( Voosen, 2017). More than 20 years ago, EGU introduced the unique concept of open discussion and transparent peer review in which preprints were posted online; they now have a centralized preprint service EGUsphere.

**Figure 1.**
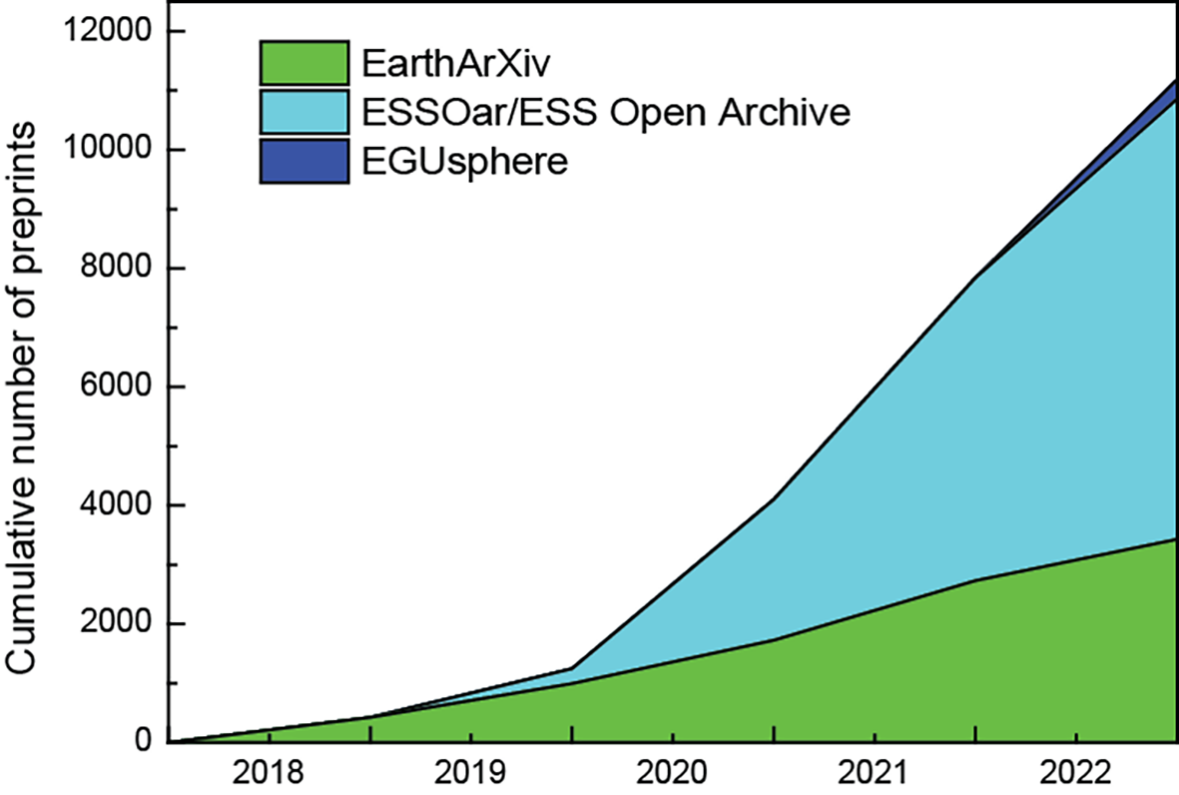
Cumulative numbers of preprints from EarthArXiv, ESS Open Archive and EGUsphere (data sourced from preprint servers individually, accessed on January 02 2023).

As illustrated on Figure 1, the cumulative numbers of preprints from EarthArXiv, ESSOAr/ESS Open Archive and EGUsphere increased this last five past years; EarthArXiv published 3,429 preprints in five years, ESSOAr/ESS Open Archive published 7,436 in four years and EGUsphere published 326 preprints in less than a year (see Table 1 for details). These numbers still continue to grow and are following a similar track that preprints in biomedical disciplines did ten years ago ( Penfold and Polka, 2019) but are not exponential as in medicine during COVID-19 pandemic ( Watson, 2022).

**Table 1.** Number of preprints by preprint server by year (data sourced from preprint servers individually, accessed on January 02 2023).

	2018	2019	2020	2021	2022
EarthArXiv	425	570	731	1006	697
ESSOAr/ESS Open Archive	-	253	2123	2738	2322
EGUsphere	-	-	-	-	326

Some other regional preprint services also exist as well as more general ones (e.g. Irawan *et al*., 2022); a list can be found here ( Kirkham *et al*., 2020).

## Benefits and issues using preprints

Preprints have numerous, well-established advantages for both researchers and the general audience (e.g., Bourne *et al*., 2017; Sarabipour *et al*., 2019; Pourret and Irawan, 2022). It is the author’s opinion that preprints, for instance, allow:

• The quick dissemination of research findings, which is important for time-sensitive studies (such as those conducted after natural disasters), for early-career researchers (ECRs) applying for jobs, or for any academic applying for grants or a promotion, given that journal-led peer review can take months or even years ( Nguyen *et al.*, 2015);
• Increased visibility and accessibility for research outputs due to the preprint’s free uploading and viewing, especially for individuals who do not have access to paywalled journals or who have restricted access because of remote working (such as during lockdowns);
• Increased visibility may also lead to increased interdisciplinary or transdisciplinary work in fields that would benefit from collaboration between Earth scientists and other disciplines (e.g., Dwivedi *et al*., 2022). Examples include geologic carbon dioxide removal strategies, water resources management and critical minerals.
• Peer feedback that goes above and beyond what is offered through journal-led peer review ( Tennant and Ross-Hellauer, 2020), increasing the likelihood of collaboration through community input and discussion; ECRs can also trained and write their first peer-review of preprints without being asked to.
• Researchers to set priority (or a precedent) for their findings to reduce the possibility of being "scooped" by being assigned a digital object identifier (DOI). Some researchers may be afraid or unable to present their results at conferences. Additionally, abstracts available in conference books and proceedings might not always reflect what is presented on the day of the conference. Preprints allow research output to exist, be known and be stored in the digital world;
• Dismantling of silos that traditional journals sustain by exposing us to a wider range of research than we might otherwise encounter and providing a home for works that do not clearly have a traditional peer-review publication as their intended destination ( *i.e.* sharing diverse types of outputs such as data, research code, or methods);
• Openness and transparency in research, with a focus on enhancing the overall standard, reliability, and reproducibility of findings.

Despite these benefits, some authors point out that preprints without peer review raise a host of issues that may vary by discipline and publication type (e.g. Meinert, 2020). In particular, they may come with a caveat that interpretations are subject to change and that they may or may not lead to actual peer reviewed publication. Pourret *et al*. (2020) pointed out that the increased dissemination effect has the potential to be used to promote non-reproducible scholarship or fake news and adds an extra potential burden on readers. But fake news has plagued climate and environmental science for decades (e.g. Nature Communications Editorial Board, 2017) and it is not specific to just preprinted papers. Preprints may have some other disadvantages, including information overload, insufficient quality assurance, political influence, and outsized impact (e.g. Smart, 2022).

Posting preprints is advantageous for ECRs because they can be shared, cited, and demonstrate productivity. However, the decision to preprint a manuscript must be made by all of the co-authors, and ECRs are frequently not the decision-maker due to power dynamics associated with academia ( Ettinger *et al.*, 2022). As a result, ECRs could encounter circumstances in which they are eager to deposit a preprint but are unsure of how to contact their co-authors or bring up the possibility of preprinting to their advisors. It is especially important for those of them leaving their research group after a contractual term. Indeed, in a short time it is not always possible to fully write a research paper in this particular field, as the process of conducting a field study, sampling and geochemical analyses could take years.

Based on policies collated on Sherpa Romeo of the earth sciences journals *,* a majority of those journals do accept manuscripts preprinted prior to or during submission. As an example 84% of journals in geochemistry allow for preprinting ( Pourret *et al*., 2020). The journals that do not offer a preprint option often do that because their thematic articles are mostly invited, generally review papers, and very rarely include the release of new data. This discrepancy is an example where the style and purpose of a given journal or magazine may influence editors and editorial boards to treat preprints differently based on the objectives of that scientific publication.

## Concluding remarks

Overall, preprints have played a crucial role in advancing science for the benefit of humanity during the pandemic, according to the opinions of medical and scientific communities as well as the general people ( Besançon *et al*., 2021). They are now included in some major bibliographic databases. Even if not always allowed by some funding agencies (e.g. Australian Research Council, Lanati *et al*., 2021), preprints are now a recognized step in the publication of scientific research and will continue to be used. For example, on Open Research Europe, the open access platform of Horizon 2020, Horizon Europe and Euratom funded projects, submitted articles are published prior to peer review, similar to preprints. Indeed, preprints are assisting in the modernization of our disciplines by reducing structural hurdles that prevent taxpayers, who frequently support knowledge development, from accessing science and knowledge, as well as by making research findings rapidly available to anybody who might benefit from them. The preprint landscape is moving fast, in early December 2022 *PLOS* announced in a press release a new partnership with *EarthArXiv.*

Additionally, *PLOS* , in partnership with *DataSeer* , has just released the first Open Science Indicators dataset, which uses large-scale Natural Language Processing to analyze published research articles to identify and track Open Science practices ( Public Library of Science, 2022). The first three indicators included are: data sharing, code sharing, and preprint posting. Importantly, these metrics are not intended to rate or rank journals or publishers, but rather to set benchmarks, monitor changes over time, and better understand the research community’s use of Open Science practices such as preprinting. Even if bioRxiv reports up to 53% of preprints that are later published as papers ( Abdill and Blekhman, 2019), Eckmann and Bandrowski (2023) estimated a bigger conversion from preprints to published articles. It is the author’s opinion that preprints are certainly here to stay!

## Data Availability

No data are associated with this article.
