# Supplementary material for: The rise of preprints in earth sciences
Source: F1000Res. 2023 Jul 10;12:561. Originally published 2023 May 30. [Version 2] doi: 10.12688/f1000research.133612.2 (PMC10336359; doi:10.12688/f1000research.133612.2)
Supplement: Peer review history

# Peer review: Reviewer response for version 2

**DOI:** 10.5256/f1000research.152594.r185628

**Published:** 2023-07-21

**Authors:** Blanca Rodriguez-Bravo

**Affiliations:** 1 Department of Library and Information Science, University of León, León, Castile and León, Spain

I congratulate the authors for the new version of the article that is more complete with the information added to the introduction about the preprint servers, mainly. Besides, I thank them for the full consideration of my comments. I can see the issues of using preprints more complete and both benefits and issues better balanced.

Is the topic of the opinion article discussed accurately in the context of the current literature?

Yes

Are arguments sufficiently supported by evidence from the published literature?

Partly

Are all factual statements correct and adequately supported by citations?

Yes

Are the conclusions drawn balanced and justified on the basis of the presented arguments?

Yes

Reviewer Expertise:

Scientific communication; Information behaviour; Knowledge organization

I confirm that I have read this submission and believe that I have an appropriate level of expertise to confirm that it is of an acceptable scientific standard.

# Peer review: Reviewer response for version 2

**DOI:** 10.5256/f1000research.152594.r185630

**Published:** 2023-07-11

**Authors:** Daniel J. Dunleavy

**Affiliations:** 1 Center for Translational Behavioral Science, Florida State University, Tallahassee, Florida, USA

I thank the authors for their consideration of my previous comments and questions. Overall, I was satisfied with the responses to the feedback from both myself and the other reviewers. 

 (1) I think the discussion of the three preprint servers in the Earth Sciences is markedly improved.

 (2) The authors say that, "Like for the scientific literature in general, the main advantages of discipline-specific servers, compared to using any number of other “generic” platforms (e.g., Zenodo or simply OSF Preprints) is to be specific to the discipline."

 I still think this point could still be fleshed out in more detail. I intuitively understand what is meant by the statement - but feel that the point could be articulated more fully.

 (3) Re: Benefits of preprints. Something that is tacitly discussed is the concept of version control. This could be unpacked just a bit more, in my opinion. While some journals and platforms (e.g., F1000) readily support version control, one of the biggest benefits of (most) preprint platforms, in my estimation, is the ability to continuously update/expand/refine/etc. - such that manuscripts become more of a living-document, rather than the "final word" on a project or subject.

Is the topic of the opinion article discussed accurately in the context of the current literature?

Yes

Are arguments sufficiently supported by evidence from the published literature?

Partly

Are all factual statements correct and adequately supported by citations?

Yes

Are the conclusions drawn balanced and justified on the basis of the presented arguments?

Partly

Reviewer Expertise:

I am an academic social worker by training (PhD) with an extensive record of publishing on issues related to open science, peer review, and issues in scholarly publishing.

I confirm that I have read this submission and believe that I have an appropriate level of expertise to confirm that it is of an acceptable scientific standard.

# Peer review: Reviewer response for version 1

**DOI:** 10.5256/f1000research.146618.r175863

**Published:** 2023-06-20

**Authors:** Daniel J. Dunleavy

**Affiliations:** 1 Center for Translational Behavioral Science, Florida State University, Tallahassee, Florida, USA

The authors have written an opinion piece outlining the rise and use of preprints in the earth sciences. They briefly describe the history of preprints, identify three common preprint platforms used in the field, and articulate the strengths and limitations for researchers and scholars of posting preprints.

 I found the manuscript to fill a somewhat niche, but relevant area of discussion. Its strengths lie in its overall exposition of the strengths and limitations of preprints and overview of the *EarthArXiv, ESSOAr/ESS Open Archive,* and *EGUsphere* platforms.

 I hope the following comments, questions, and suggestions help strengthen the overall quality of the manuscript. Additionally, please be advised that any and all references included in this review are suggestive and not meant to be viewed as compulsory.

**Introduction**

 (1) I somewhat disagree with the opening sentence. Though it includes manuscripts that are best described as an "initial draft shared online", the term preprint (somewhat unfortunately) appears to encompass many different things (including post-print manuscripts and materials and resources never intended for publication). There's a brief, but decent discussion here:

https://scholarlykitchen.sspnet.org/2017/04/19/preprint-server-not-preprint-server/ (see also the comments)

 and here:

https://doi.org/10.31222/osf.io/796tu.

 You might consider adding a sentence or two acknowledging this and emphasizing whatever definition you prefer to in the context of this discussion.

 (2) Similarly, though not "necessary", the introduction might also make further mention of the history of preprints prior to *arXiv*. This might help further contextualize and possibly contrast the historical, professional, and institutional factors that (have and continue to) motivate scientists to engage with or refrain from sharing via preprint servers. 

 See: https://doi.org/10.1371/journal.pbio.2003995, https://doi.org/10.1002/meet.2014.14505101036,

 and

https://www.science.org/doi/10.1126/science.154.3751.843.a

**Preprints in the earth sciences**

 (3) This section felt particularly "thin". I think it would be strengthened by comparing and contrasting the three servers/platforms in greater detail. As a reader, I did not feel I got a good understanding how these platforms differed and what benefits one might offer over another (in terms of visibility, content moderation, indexing, tools, support, etc.).

 (4) What's more, this section might be improved by a brief discussion of the advantages of discipline-specific servers, compared to using any number of other "generic" platforms (e.g., Zenodo or simply OSF Preprints).

**Benefits and issues using preprints**

 (5) You've already cited Ginsparg, 2016 - but I think their work provides a nice (if somewhat opinionated) place for expanding and more fully elaborating upon the strengths and limitations of sharing via preprint (see FAQ9 and FAQ15, for example). 

**Data and materials**

 (6) The manuscript may benefit from another sentence or two describing how the data for Table 1 and Figure 1 were sourced. As the numbers don't appear (to me) to be readily accessible from the preprint servers, I imagine each site was searched, by year, to generate the total number of preprints. Whatever the case, a bit more explanation may be helpful for enhanced transparency and reproducibility.

**Miscellaneous comments**

 (7) I can appreciate that the manuscript was previously published as a preprint on *EarthArXiv*. If applicable, it might be useful to include a statement or two about whether and how doing so benefited its development or dissemination, if at all.

Is the topic of the opinion article discussed accurately in the context of the current literature?

Yes

Are arguments sufficiently supported by evidence from the published literature?

Partly

Are all factual statements correct and adequately supported by citations?

Yes

Are the conclusions drawn balanced and justified on the basis of the presented arguments?

Partly

Reviewer Expertise:

I am an academic social worker by training (PhD) with an extensive record of publishing on issues related to open science, peer review, and issues in scholarly publishing.

I confirm that I have read this submission and believe that I have an appropriate level of expertise to confirm that it is of an acceptable scientific standard, however I have significant reservations, as outlined above.

## References

[1] : A tale of two 'opens': intersections between Free and Open Source Software and Open Scholarship.2020; 10.31235/osf.io/2kxq8 10.31235/osf.io/2kxq8

[2] : When is a preprint server not a preprint server?. *The Scholarly Kitchen* .2017, April 19; Reference source

[3] : The evolving preprint landscape: Introductory report for the Knowledge Exchange working group on preprints. 2018; 10.31222/osf.io/796tu 10.31222/osf.io/796tu

[4] : The prehistory of biology preprints: A forgotten experiment from the 1960s. *PLoS Biol* .2017;15(11) : 10.1371/journal.pbio.2003995 e2003995 10.1371/journal.pbio.2003995 29145518PMC5690419

[5] : Enclaves of anarchy: Preprint sharing, 1940-1990. *Proceedings of the American Society for Information Science and Technology* .2014;51(1) : 10.1002/meet.2014.14505101036 1-10 10.1002/meet.2014.14505101036

[6] : Information Exchange Groups To Be Discontinued. *Science* .1966;154(3751) : 10.1126/science.154.3751.843.a 10.1126/science.154.3751.843.a 6003532

[7] : Preprint Déjà Vu. *EMBO J* .2016;35(24) : 10.15252/embj.201695531 2620-2625 10.15252/embj.201695531 27760783PMC5167339

# Response

**Published:** 2023-07-04

**Authors:** Olivier Pourret

**Affiliations:** AGHYLE, UniLaSalle, Beauvais, Hauts-de-France, France

Thank you for all your comments and suggestions. We have reworked our article as follows.

- We have reworked the first paragraph and added some nuances as proposed by reviewer 3. “Though it includes manuscripts that are best described as an "initial draft shared online", the term preprint (somewhat unfortunately) appears to encompass many different things (including post-print manuscripts and materials and resources never intended for publication) (see discussion in Tennant *et al.*, 2018). “
- We have added some details in the introduction: “In 1961, the USA National Institutes of Health launched a program called Information Exchange Groups, designed for the circulation of biological preprints, but this was shut down in 1967 (Confrey, 1966; Cobb, 2017).”
- As also suggested by reviewer 2,  we have added details on the 3 preprint servers mentioned in our submission. 
- We have added some details of the advantages of discipline-specific servers, compared to using any number of other "generic" platforms.
- We have added some details on Benefits and issues using preprints and further cite the work of Ginsparg (2016).
- We have added a sentence on the methodology on how the numbers were generated.
- We have added some words in the acknowledgment section stating that our previous preprint at EarthArXiv let us being invited to submit this opinion paper at F1000Research.

# Peer review: Reviewer response for version 1

**DOI:** 10.5256/f1000research.146618.r175864

**Published:** 2023-06-20

**Authors:** Zhiqi Wang

**Affiliations:** 1 WISE Lab, Dalian University of Technology, Dalian, Liaoning, China

The opinion paper offers insights into the rise of preprints in earth sciences, focusing on three main preprint servers: EarthArXiv, ESSOAr/ESS Open Archive, and EGUsphere. The authors highlight an increasing trend in the cumulative numbers of preprints posted on these servers, indicating the growing popularity of preprints in the field. They further discuss the benefits and issues associated with preprint publishing.

 To enhance the overall content, I would suggest the following improvements:

1. Regarding the first research problem addressed, the trend in preprint publishing, I suggest the authors to consider additional factors beyond cumulative numbers. In my opinion, factors such as submission rate, citations, and social attention attracted by the preprints can provide a more comprehensive and reliable assessment of the trend.
2. For the three preprint servers mentioned, I think it would be valuable to outline their distinct features. This information would help readers understand how to choose the most suitable server for posting or searching preprints.
3. As an opinion paper, I suggest the authors to give more further discussion on the specific benefits and challenges unique to preprint publishing in the field of earth sciences, as compared to other disciplines. Considering that the readers of the paper likely come from the field, addressing this topic would provide them with valuable insights. And in the concluding remarks section, the paper will benefit for including comments on the future development of preprints in the field of earth sciences.

Is the topic of the opinion article discussed accurately in the context of the current literature?

Yes

Are arguments sufficiently supported by evidence from the published literature?

Partly

Are all factual statements correct and adequately supported by citations?

Yes

Are the conclusions drawn balanced and justified on the basis of the presented arguments?

Yes

Reviewer Expertise:

Scientometrics, science of science, scientific communication, preprint, S&T management

I confirm that I have read this submission and believe that I have an appropriate level of expertise to confirm that it is of an acceptable scientific standard, however I have significant reservations, as outlined above.

# Response

**Published:** 2023-07-04

**Authors:** Olivier Pourret

**Affiliations:** AGHYLE, UniLaSalle, Beauvais, Hauts-de-France, France

Thank you for your comments, we have refined our article following your suggestions.

- The comment is very pertinent, unfortunately we do not have access to data like submission rate, citations, and social attention attracted by the preprints. Certain publishers linked to preprint servers (e.g., AGU journals published in Wiley linked to ESSOAr/ESS Open Archive) would be able to collate such data, but that is outside the scope of our analysis here.
- Agreed, we have added details in the form of short paragraphs on each of the 3 preprint servers mentioned in our submission.  
- We have added a few more examples dedicated to Earth Sciences and added a sentence on future development of preprints in our discipline in conclusion. Thank you again for all your comments.

# Peer review: Reviewer response for version 1

**DOI:** 10.5256/f1000research.146618.r175861

**Published:** 2023-06-15

**Authors:** Blanca Rodriguez-Bravo

**Affiliations:** 1 Department of Library and Information Science, University of León, León, Castile and León, Spain

Congratulations to the authors for this paper, that is relevant. This is an opinion article that begins by defining and briefly discussing the history of preprints and then focuses on the evolution of three preprint repositories in the field of earth sciences. The article provides quantitative data that confirms the growing interest in the aforementioned platforms, and then addresses the advantages and disadvantages of publishing through preprints. The information about the three preprints platforms is interesting.

 The authors' vision seems adequate to me, although I believe that in the search for greater symmetry the issues should be presented as the benefits -by means of bullets and with a more complete argumentation-. My view is that benefits are clearly and completely highlighted but this is not the case of the drawbacks. The problems derived by a less formal or less complete peer review process may be expanded as it is the case of information overload, insufficient quality assurance, or non-reproducible research. The main problem can be that the reader is not aware that he/she may have been reading a paper that no one has evaluated yet. I am not familiar with the field of earth sciences but in general there is still a high lack of knowledge about preprints as some of them are eventually published in journals and others are not.

 In the case of Early Career Researchers (ECRs), to which the authors pay special attention, it is true that the quick dissemination of research findings is a clear advantage for ECRs. However, they are also wary of publications that do not pass prior quality control. Therefore, they often hold back from publishing preprints, and not just because coauthors, senior researchers, prefer to publish in traditional journals. ECRs, due to their competitive circumstances, are sometimes more opposed to publishing preprints that do not lead to a traditional publication than other already tenured researchers. Check, for instance, the paper by Nicholas et al. (2022) published in Profesional de la información, v.31, n.4, e310418. 

 The conclusions are appropriate. Open science  is a necessity and preprints, as one of its manifestations, are growing in importance as both the subscription model of journals and the APCs model are more and more questioned.

 To sum up, although the paper is well structured and well argued, the paper will benefit for expanding the "still" negative aspects of preprints taking into account the points of view of authors -with particular attention to ECRs- and readers.

Is the topic of the opinion article discussed accurately in the context of the current literature?

Yes

Are arguments sufficiently supported by evidence from the published literature?

Partly

Are all factual statements correct and adequately supported by citations?

Yes

Are the conclusions drawn balanced and justified on the basis of the presented arguments?

Yes

Reviewer Expertise:

Scientific communication; Information behaviour; Knowledge organization

I confirm that I have read this submission and believe that I have an appropriate level of expertise to confirm that it is of an acceptable scientific standard, however I have significant reservations, as outlined above.

## References

[1] : Early career researchers in the pandemic-fashioned ‘new scholarly normality’: a first look into the big changes and long-lasting impacts (international analysis). *El Profesional de la información* .2022; 10.3145/epi.2022.jul.18 10.3145/epi.2022.jul.18

# Response

**Published:** 2023-07-04

**Authors:** Olivier Pourret

**Affiliations:** AGHYLE, UniLaSalle, Beauvais, Hauts-de-France, France

Thank you for your comments and have reworked our article following your suggestions.

- We have expanded the drawbacks section (see next response as well).
- We have added a bullet and have considered the proposed reference: “ECR may wary of publications that do not pass prior quality control. Therefore, they often hold back from publishing preprints, and not just because coauthors, senior researchers, prefer to publish in traditional journals. ECRs, due to their competitive circumstances, are sometimes more opposed to publishing preprints that do not lead to a traditional publication than other already tenured researchers (Nicholas et al., 2022)”

Overall, we have refined our article as proposed and expanded the negative aspects of preprints, thank you again.
